# Personalized Mortality Prediction Driven by Electronic Medical Data and a Patient Similarity Metric

**DOI:** 10.1371/journal.pone.0127428

**Published:** 2015-05-15

**Authors:** Joon Lee, David M. Maslove, Joel A. Dubin

**Affiliations:** 1 School of Public Health and Health Systems, University of Waterloo, Waterloo, Ontario, Canada; 2 Department of Medicine & Critical Care Program, Queen’s University, Kingston, Ontario, Canada; 3 Department of Statistics and Actuarial Science, University of Waterloo, Waterloo, Ontario, Canada; Queen's University Belfast, UNITED KINGDOM

## Abstract

**Background:**

Clinical outcome prediction normally employs static, one-size-fits-all models that perform well for the average patient but are sub-optimal for individual patients with unique characteristics. In the era of digital healthcare, it is feasible to dynamically personalize decision support by identifying and analyzing similar past patients, in a way that is analogous to personalized product recommendation in e-commerce. Our objectives were: 1) to prove that analyzing only similar patients leads to better outcome prediction performance than analyzing all available patients, and 2) to characterize the trade-off between training data size and the degree of similarity between the training data and the index patient for whom prediction is to be made.

**Methods and Findings:**

We deployed a cosine-similarity-based patient similarity metric (PSM) to an intensive care unit (ICU) database to identify patients that are most similar to each patient and subsequently to custom-build 30-day mortality prediction models. Rich clinical and administrative data from the first day in the ICU from 17,152 adult ICU admissions were analyzed. The results confirmed that using data from only a small subset of most similar patients for training improves predictive performance in comparison with using data from all available patients. The results also showed that when too few similar patients are used for training, predictive performance degrades due to the effects of small sample sizes. Our PSM-based approach outperformed well-known ICU severity of illness scores. Although the improved prediction performance is achieved at the cost of increased computational burden, Big Data technologies can help realize personalized data-driven decision support at the point of care.

**Conclusions:**

The present study provides crucial empirical evidence for the promising potential of personalized data-driven decision support systems. With the increasing adoption of electronic medical record (EMR) systems, our novel medical data analytics contributes to meaningful use of EMR data.

## Introduction

Patient outcome prediction has been identified as one of the key learning applications of big health care data [1], and plays important roles in clinical medicine as it is tightly related to intervention selection, care planning, and resource allocation. Traditionally, clinical prognostication has relied on static models generated from analyzing large, heterogeneous, multi-center patient datasets. For example, severity of illness (SOI) scores used in intensive care, such as the Acute Physiology and Chronic Health Evaluation (APACHE) [2] or Simplified Acute Physiology Score (SAPS) [3] systems, were developed based on large-scale data collected from numerous countries. Although such one-size-fits-all approaches perform well for the average patient, how well they perform for patients whose characteristics deviate from the average patient is questionable.

Case-mix has been a major challenge for clinical prognostication; different SOI scores have been shown to result in substantial prognostic disagreement for the same patient populations [4]. As a result, customized predictive modeling using local data has seen increased research activity; e.g., Celi et al. have shown that customized models can lead to better mortality prediction performance for specific patient cohorts [5]. In addition, a recent report from MIT Technology Review described the general enthusiasm around harnessing the personalized information in big health data collected from electronic medical records (EMRs), genome sequencing, and ubiquitous environmental and behavioral monitoring [6].

While personalized data-driven prediction in clinical medicine is still a developing field, this kind of data analytic paradigm has been applied successfully in other domains such as future career trajectory prediction for Major League Baseball players in sports analytics [7]; personalized product recommendation in e-commerce [8]; and credit scoring used by financial services to predict the likelihood of making payments on time [9]. In particular, collaborative filtering, which is a popular technique employed by many modern recommender systems, makes predictions for a particular user by collecting preferences from other users who have shown similar preferences in the past [10,11]. One of the few attempts to date to measure patient similarity was conducted by Sun et al. who utilized supervised machine learning and clinician input to define a distance-based similarity measure [12]. All of these applications extracted useful predictive information from similar past cases.

We hypothesized that as electronic capture of medical data becomes increasingly commonplace, clinical outcome prediction can become personalized—and more precise as a result—by identifying and analyzing past patients who were similar to a present case of interest (index patient), whose outcome is to be predicted. The central idea behind our hypothesis is that the amount of predictive utility contributed by a past patient should be directly proportional to the degree of similarity between the past and index patient. The converse of this argument is that data from dissimilar patients may actually degrade predictive performance since they are primarily irrelevant to the index patient. We also hypothesized that the improvement in predictive performance achieved by focusing on similar patients would be limited if the number of similar patients decreases too much. This would occur if stringent patient similarity criteria were applied, since the limitations of smaller sample sizes would offset the positive effects of increased personalization.

The core foundation of the proposed personalized outcome prediction is a patient similarity metric (PSM) that quantifies the degree of similarity between the index patient and a past patient recorded in electronic medical data. Although various PSM definitions are conceivable (contingent on available clinical variables and patient type), we employed a simple, intuitive PSM in the present study to test our hypotheses.

Among all medical specialties, intensive care is particularly well suited for the present study because of the enormous amount and breadth of clinical data that typical intensive care units (ICUs) collect on a daily basis in order to closely monitor fragile ICU patients [13]. Granular ICU data enable detailed patient similarity matching. Furthermore, improved clinical outcome prediction can facilitate optimized resource allocation and real-time decision making in the ICU, which is an important issue to address given the increasing demand for intensive care in many countries with ageing populations [14].

The objectives of the present study were: 1) to prove that mortality prediction can be improved by using training data extracted only from a subset of similar patients in comparison with all available patients, and 2) to characterize the relationship between the number of most similar patients used for training and the extent of similarity between the index patient and the past patients in the training data, with respect to mortality prediction performance.

## Methods

### Patient data extraction

The patient data for this study were extracted from the MIMIC-II database [15]. MIMIC-II is publicly available and contains data from 29,149 adult ICU admissions (version 2.6) at the Beth Israel Deaconess Medical Center (BIDMC) in Boston, MA. MIMIC-II is a one-of-a-kind database with rich clinical data including vital signs every 10–15 minutes, lab test results 1–4 times a day, and hourly urine output measurements. MIMIC-II also contains International Classification of Diseases 9 (ICD-9) codes, daily SAPS [3] and Sequential Organ Failure Assessment [16] scores, radiology reports, nursing notes, discharge summaries, ICU and hospital lengths of stay, and out-of-hospital mortality data. MIMIC-II data were collected from the following specialty services: medical ICU (MICU), surgical ICU (SICU), coronary care unit (CCU), and cardiac surgery recovery unit (CSRU). Because MIMIC-II is a public, de-identified database, the need to obtain informed consent or research ethics approval has been waived.

For predictor variables, various clinical and administrative variables were extracted from MIMIC-II from each ICU admission. First, the minimum and maximum values of the following vital signs were extracted from each non-overlapping 6-hour period during the first 24 hours in the ICU (i.e., each 6-hour period yielded a separate predictor): heart rate, mean blood pressure, systolic blood pressure, SpO_2_, spontaneous respiratory rate, and body temperature. Second, the minimum and maximum values of the following lab variables were extracted from the first 24 hours in the ICU: hematocrit, white blood cell count, serum glucose, serum HCO_3_, serum potassium, serum sodium, blood urea nitrogen, and serum creatinine. Minimum and maximum values were extracted for vital signs and lab test results since either could be the worst value that usually contains useful predictive information (e.g., SAPS uses maximum and minimum values [3]). Third, the following categorical variables were extracted: admission type (elective, urgent, emergency), gender, ICU service type (MICU, SICU, CCU, CSRU), primary ICD-9 code, the receipt of vasopressor therapy during the first 24 hours in the ICU (binary), and the use of mechanical ventilation or Continuous Positive Airway Pressure during the first 24 hours in the ICU (binary). Lastly, the following predictors were also extracted: age, the minimum (i.e., worst) Glasgow Coma Scale, and the total urinary output from each non-overlapping 6-hour period during the first 24 hours in the ICU.

As the clinical outcome of interest, mortality at 30 days post-discharge from the hospital, represented as a binary variable, was extracted. In addition, SAPS and SOFA scores from the first day in the ICU were also extracted for benchmarking purposes.

Only the ICU admissions with complete data were included in this study. Each ICU admission was treated as a separate patient; no check was done to distinguish ICU admissions from the same patient. Not only that most patients in MIMIC-II have only one ICU admission each (1.24 ICU admissions per patient on average), but also the rationale was to objectively rely on clinical similarity and identify similar clinical cases regardless of patient identity. Hence, ICU admissions are referred to as “patients” throughout this article.

All data were extracted from MIMIC-II using Structured Query Language (SQL) in Oracle SQL Developer (version 3.2.09).

### Patient similarity metric

Each patient was represented as a Euclidean vector in the multi-dimensional feature space defined by the predictor variables described in the previous section. The PSM in the present study was defined as the cosine of the angle between two patient vectors, the calculation of which can be facilitated by the dot product between them. This is called cosine similarity, and is widely used in text mining [17]. Hence, the PSM was mathematically defined as follows:

$$PSM(P_{1},P_{2})=\frac{P_{1}\cdot P_{2}}{\left\|P_{1}\right\|\left\|P_{2}\right\|},$$

where **P**_**1**_ and **P**_**2**_ are the predictor vectors corresponding to two different patients, while and || || represent the dot product and Euclidean vector magnitude, respectively. Because this PSM is the cosine of an angle, it is normalized between -1 (minimum similarity) and 1 (maximum similarity). Two vectors in exactly opposite directions (i.e., 180° between them) would result in a PSM value of -1, whereas two identical vectors (i.e., 0° between them) would yield a PSM value of 1.

Prior to PSM calculation, each continuous predictor was normalized to fit the range between -1 and 1 so that all predictors could equally contribute to the PSM. For each categorical predictor, the product between two vectors in that particular dimension was assigned a value of 1 if the two vectors had the same category and a value of -1 if the two vectors had different categories, in an all-or-none fashion.

### Predictive model training, evaluation, and comparisons

Three types of predictive models were deployed in 10-fold cross-validation: death counting (simply using the mortality rate among similar patients as the prediction), logistic regression (LR), and decision tree (DT). For each patient in the test data as the index patient, the following steps were executed:

1. All pairwise PSM values between the index patient and every patient in the training data were calculated.
2. The calculated PSM values were sorted in descending order.
3. Data from the N most similar patients were used to train the three predictive models, where N was varied from 10 to 500 with a step size of 10 for death counting, from 5000 to all patients in the training data with a step size of 1000 for LR, and from 2000 to all patients in the training data with a step size of 1000 for DT. In other words, only the data from the N most similar patients were utilized as training data for the given index patient.
4. Each custom-trained model predicted the mortality risk of the index patient by yielding a number between 0 and 1.

By trial and error, 5000 was determined to be the minimum number of similar patients for LR to ensure sufficient variability in categorical predictors within training data (i.e., LR requires at least one example of each category in training data to estimate all beta coefficients). Similarly, 2000 was set as the minimum for DT to ensure variability in the mortality outcome; unlike LR, DT can decide to ignore categorical predictors with negligible predictive utility due to insufficient variability. These minimum numbers of similar patients may change for different datasets and predictors.

For each of the 10 cross-validation folds, predictive performance was evaluated by calculating the area under the receiver operating characteristic curve (AUROC) as well as the area under the precision-recall curve (AUPRC). AUPRC is an informative performance measure for binary classification and complements AUROC for skewed datasets such as the one investigated in this study (the overall mortality rate was much less than 50%) [18]. This is because precision, also known as positive predictive value, is naturally low for datasets with relatively rare positive cases (i.e., low prevalence), while a high AUROC can be achieved by focusing on the majority negative cases. It has been formally shown that predictive models that optimize AUROC do not necessarily optimize AUPRC [18]. Similarly to AUROC, AUPRC values range from zero to one, where one indicates perfect prediction; however, random guessing does not achieve an AUPRC of 0.5 for skewed datasets. In order to mitigate the effects of the skewed dataset in this study, the 10-fold cross-validation incorporated stratified sampling to ensure that the ratio between the positive (expired) and negative (survived) cases in each fold was similar to that in the entire dataset. In other words, the dataset was first divided into two strata: the expired and survived. Then, random assignment to the 10 cross-validation folds was carried out in each stratum independently.

For benchmarking, the predictive performances (i.e., AUROC and AUPRC) of SAPS and SOFA were also quantified on the same cross-validation data partitions. Both SAPS and SOFA scores were customized by using each as the only predictor in an LR model (i.e., customized via logit-transformation [19]). This means that mortality was regressed on either the raw SAPS or SOFA scores, in a univariable LR model, to find the beta coefficient fitted to the training data. All training data, rather than just N similar patients, were used in this customization.

For each predictive model, the peak performance, in terms of either AUROC or AUPRC, was compared with the performance associated with the maximum number of patients, as well as with the SAPS and SOFA performances. These comparisons were conducted via two-sided two-sample t-tests with a significance level of alpha = 0.05.

All computations and analyses were conducted in R (version 3.1.1).

## Results

### Patient data

A total of 17,152 ICU admissions in the MIMIC-II database had complete data and were included in the study. Table 1 summarizes the patient data in terms of several key clinical and administrative variables. The overall 30-day mortality rate was 15.1%.

**Table 1:** Patient data characteristics.

Number of unique ICU admissions	17,152
**Admission Type (%)**	
Elective	18.0
Urgent	3.7
Emergency	78.3
**Top 5 Primary ICD-9 Codes (%)**	
414.01—of native coronary artery	10.8
410.71—subendocardial infarction	4.7
038.9—unspecified septicemia	3.6
424.1—aortic valve disorders	2.9
518.81—acute respiratory failure	2.6
**Gender (male %)**	56.7
**Age (years)**	64.5 [17.0]
**Vasopressor Therapy (%)**	36.4
**Mechanical Ventilation or CPAP (%)**	58.1
**SAPS**	14.5 [5.1]
**SOFA**	6.3 [3.9]
**30-day mortality (%)**	15.1

Age, SAPS, and SOFA are shown in mean [standard deviation]. SAPS: Simplified Acute Physiology Score; SOFA: Sequential Organ Failure Assessment.

### Predictive performances of SAPS and SOFA

To benchmark the personalized predictive models, the predictive performances of SAPS and SOFA were quantified in 10-fold cross-validation. SAPS achieved a mean AUROC of 0.658 (95% confidence interval (CI): [0.648, 0.668]) and a mean AUPRC of 0.271 (95% CI: [0.253, 0.290]). SOFA achieved a mean AUROC of 0.633 (95% CI: [0.624, 0.642]) and a mean AUPRC of 0.273 (95% CI: [0.253, 0.292]).

### Death counting among similar patients

**Fig 1:**
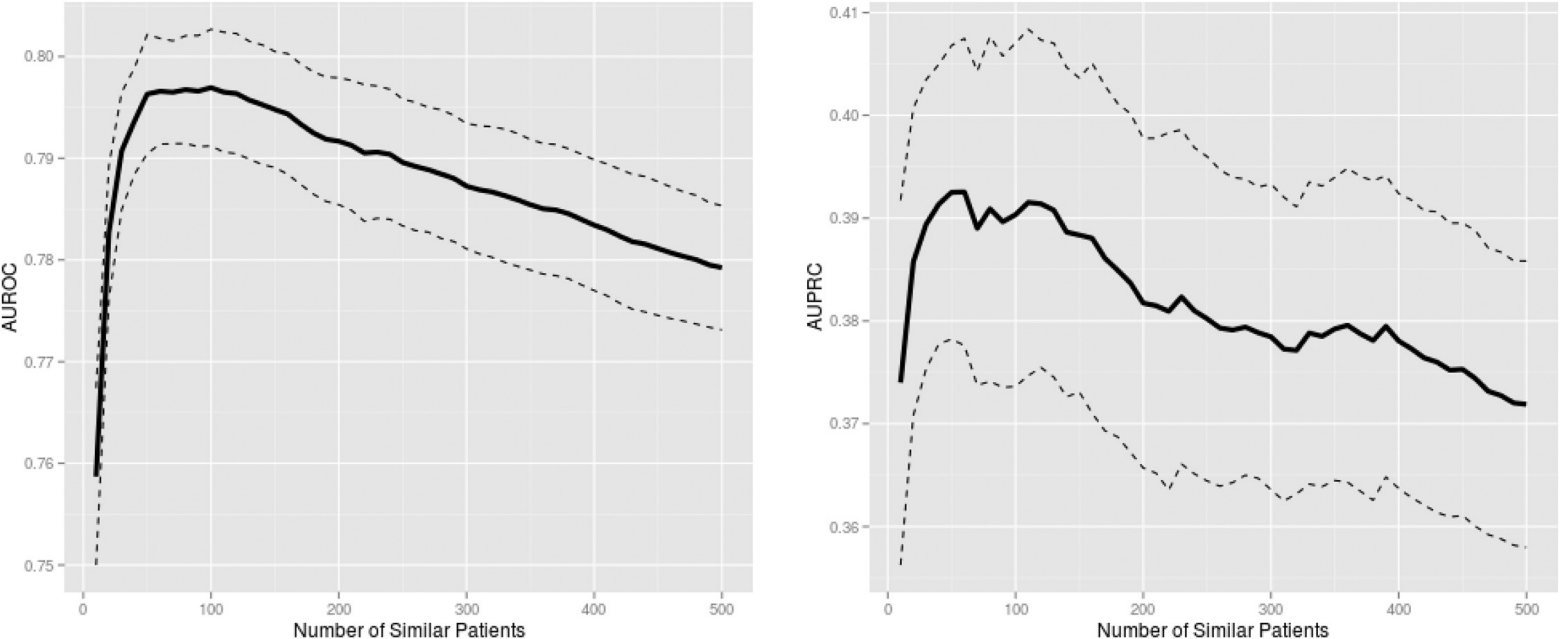
Mortality prediction performance of death counting among similar patients. The solid and dashed lines are the mean and 95% confidence intervals, respectively, from 10-fold cross-validation. A trade-off between training data homogeneity and size is apparent; as the number of similar patients in the training data increases, predictive performance improves initially at a rapid rate thanks to increasing training data size but starts to degrade gradually due to decreasing homogeneity within the training data. AUROC: area under the receiver operating characteristic curve; AUPRC: area under the precision-recall curve.

Fig 1 illustrates the AUROC and AUPRC of death counting as a function of the number of similar patients used as training data. Table 2 tabulates the results shown in Fig 1. The shown trend confirms our hypothesis that predictive performance improves as dissimilar patients are excluded from training (moving left on the X-axes in Fig 1, from 500 to roughly 100). Performance degrades again when too few patients are used for training (the left side of the peaks in Fig 1, moving left on the X-axes from 100 to 10), which again confirms our hypothesis.

**Table 2:** Mortality prediction performance of death counting as a function of the number of similar patients used in training.

Number of Similar Patients	AUROC (Mean [95% CI])	AUPRC (Mean [95% CI])
10	0.759 [0.750, 0.767]	0.374 [0.356, 0.392]
20	0.783 [0.776, 0.789]	0.386 [0.371, 0.401]
30	0.791 [0.785, 0.796]	0.389 [0.375, 0.403]
40	0.794 [0.788, 0.799]	0.391 [0.378, 0.405]
50	0.796 [0.790, 0.802]	0.393 [0.378, 0.407]
60	0.797 [0.791, 0.802]	0.393 [0.378, 0.407]
70	0.796 [0.791, 0.802]	0.389 [0.374, 0.404]
80	0.797 [0.791, 0.802]	0.391 [0.374, 0.408]
90	0.797 [0.791, 0.802]	0.390 [0.374, 0.406]
100	0.797 [0.791, 0.803]	0.390 [0.374, 0.407]
110	0.796 [0.791, 0.802]	0.392 [0.375, 0.408]
120	0.796 [0.790, 0.802]	0.391 [0.375, 0.407]
130	0.796 [0.790, 0.802]	0.391 [0.375, 0.407]
140	0.795 [0.789, 0.801]	0.389 [0.373, 0.405]
150	0.795 [0.789, 0.800]	0.388 [0.373, 0.404]
160	0.794 [0.788, 0.800]	0.388 [0.371, 0.405]
170	0.793 [0.787, 0.799]	0.386 [0.369, 0.403]
180	0.792 [0.786, 0.799]	0.385 [0.369, 0.401]
190	0.792 [0.786, 0.798]	0.384 [0.367, 0.400]
200	0.792 [0.785, 0.798]	0.382 [0.366, 0.398]
210	0.791 [0.785, 0.798]	0.381 [0.365, 0.398]
220	0.791 [0.784, 0.797]	0.381 [0.364, 0.398]
230	0.791 [0.784, 0.797]	0.382 [0.366, 0.399]
240	0.790 [0.784, 0.797]	0.381 [0.365, 0.397]
250	0.790 [0.783, 0.796]	0.380 [0.364, 0.396]
260	0.789 [0.783, 0.796]	0.379 [0.364, 0.395]
270	0.789 [0.783, 0.795]	0.379 [0.364, 0.394]
280	0.788 [0.782, 0.795]	0.379 [0.365, 0.394]
290	0.788 [0.782, 0.794]	0.379 [0.365, 0.393]
300	0.787 [0.781, 0.793]	0.378 [0.364, 0.393]
310	0.787 [0.781, 0.793]	0.377 [0.362, 0.392]
320	0.787 [0.780, 0.793]	0.377 [0.363, 0.391]
330	0.786 [0.780, 0.793]	0.379 [0.364, 0.394]
340	0.786 [0.779, 0.792]	0.379 [0.364, 0.393]
350	0.785 [0.779, 0.792]	0.379 [0.364, 0.394]
360	0.785 [0.779, 0.791]	0.380 [0.364, 0.395]
370	0.785 [0.778, 0.791]	0.379 [0.363, 0.394]
380	0.785 [0.778, 0.791]	0.378 [0.363, 0.394]
390	0.784 [0.778, 0.790]	0.379 [0.365, 0.394]
400	0.783 [0.777, 0.790]	0.378 [0.364, 0.392]
410	0.783 [0.776, 0.789]	0.377 [0.363, 0.392]
420	0.782 [0.776, 0.789]	0.376 [0.362, 0.391]
430	0.782 [0.775, 0.788]	0.376 [0.361, 0.391]
440	0.782 [0.775, 0.788]	0.375 [0.361, 0.390]
450	0.781 [0.775, 0.788]	0.375 [0.361, 0.390]
460	0.781 [0.774, 0.787]	0.374 [0.360, 0.389]
470	0.780 [0.774, 0.787]	0.373 [0.359, 0.387]
480	0.780 [0.774, 0.786]	0.373 [0.359, 0.387]
490	0.780 [0.773, 0.786]	0.372 [0.358, 0.386]
500	0.779 [0.773, 0.785]	0.372 [0.358, 0.386]

The results shown in Fig 1 are tabulated here. AUROC: area under the receiver operating characteristic curve; AUPRC: area under the precision-recall curve; CI: confidence interval.

The maximum mean AUROC of 0.797 was achieved with 100 most similar patients, while the maximum mean AUPRC of 0.393 occurred at 60 most similar patients. The peak AUROC was significantly better than the AUROC associated with the maximum number of similar patients considered, i.e., 500 (p = 0.001), but there was no significant difference in AUPRC between the peak and 500 most similar patients (p = 0.074). The peak performances were significantly better than the SAPS (AUROC: p<10^–11^; AUPRC: p<10^–9^) and SOFA (AUROC: p<10^–9^; AUPRC: p<10^–6^) performances.

With respect to the peaks in Fig 1, predictive performance worsened rapidly as the number of similar patients decreased (moving left on the X-axes, from 100 to 10), whereas it degraded more gradually as the number of similar patients increased (moving right on the X-axes, from 100 to 500).

### Logistic regression based on similar patients

**Fig 2:**
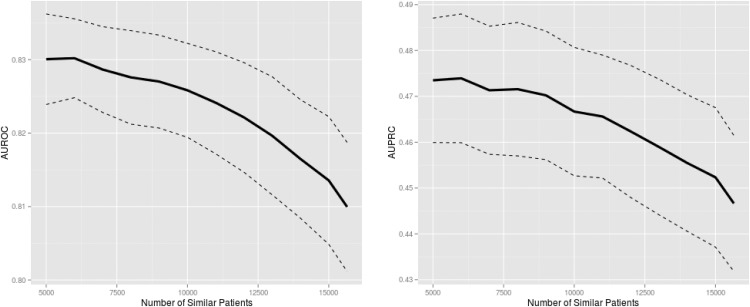
Mortality prediction performance of personalized logistic regression trained on similar patient data. The solid and dashed lines are the mean and 95% confidence intervals, respectively, from 10-fold cross-validation. The maximum number of similar patients corresponds to all available training data. Predictive performance clearly improves as data from fewer but more similar patients are used for training. Identical predictor values in training data prohibited decreasing the number of similar patients further. AUROC: area under the receiver operating characteristic curve; AUPRC: area under the precision-recall curve.

Fig 2 shows the predictive performance of personalized LR as a function of the number of similar patients used for training. Table 3 tabulates the results shown in Fig 2. Starting from using all training data (the maximum number of similar patients on the X-Axes in Fig 2), it is clearly shown that predictive performance improved as a smaller but more similar subset of patients was used for training (i.e., moving left on the X-axes).

**Table 3:** Mortality prediction performance of logistic regression as a function of the number of similar patients used in training.

Number of Similar Patients	AUROC (Mean [95% CI])	AUPRC (Mean [95% CI])
5000	0.830 [0.824, 0.836]	0.473 [0.460, 0.487]
6000	0.830 [0.825, 0.836]	0.474 [0.460, 0.488]
7000	0.829 [0.823, 0.834]	0.471 [0.457, 0.485]
8000	0.828 [0.821, 0.834]	0.472 [0.457, 0.486]
9000	0.827 [0.821, 0.833]	0.470 [0.456, 0.484]
10000	0.826 [0.819, 0.832]	0.467 [0.453, 0.481]
11000	0.824 [0.817, 0.831]	0.466 [0.452, 0.479]
12000	0.822 [0.815, 0.830]	0.462 [0.448, 0.477]
13000	0.820 [0.812, 0.828]	0.459 [0.444, 0.474]
14000	0.816 [0.808, 0.825]	0.455 [0.441, 0.470]
15000	0.814 [0.805, 0.822]	0.452 [0.437, 0.468]
15649	0.810 [0.801, 0.819]	0.447 [0.432, 0.461]

The results shown in Fig 2 are tabulated here. AUROC: area under the receiver operating characteristic curve; AUPRC: area under the precision-recall curve; CI: confidence interval.

The peak mean AUROC and AUPRC of 0.830 and 0.474, respectively, were achieved when 6000 most similar patients were used for training. These performance results were significantly better than those resulting from using all available training data in terms of AUROC (p = 0.014) but not AUPRC (p = 0.094). In comparison with SAPS and SOFA, the best personalized LR model also resulted in significantly greater AUROC (SAPS: p<10^–12^; SOFA: p<10^–9^) and AUPRC (SAPS: p<10^–12^; SOFA: p<10^–9^).

In terms of both AUROC and AUPRC, predictive performance seemingly plateaued near 5000 similar patients (i.e., the increase in performance with decreasing number of similar patients slowed down). Performance degraded more rapidly as the number of similar patients increased (moving right on the X-axes in Fig 2), especially when the most dissimilar patients were added (the right ends of the plots in Fig 2, moving right from 15000 on the X-axes).

### Decision tree based on similar patients

**Fig 3:**
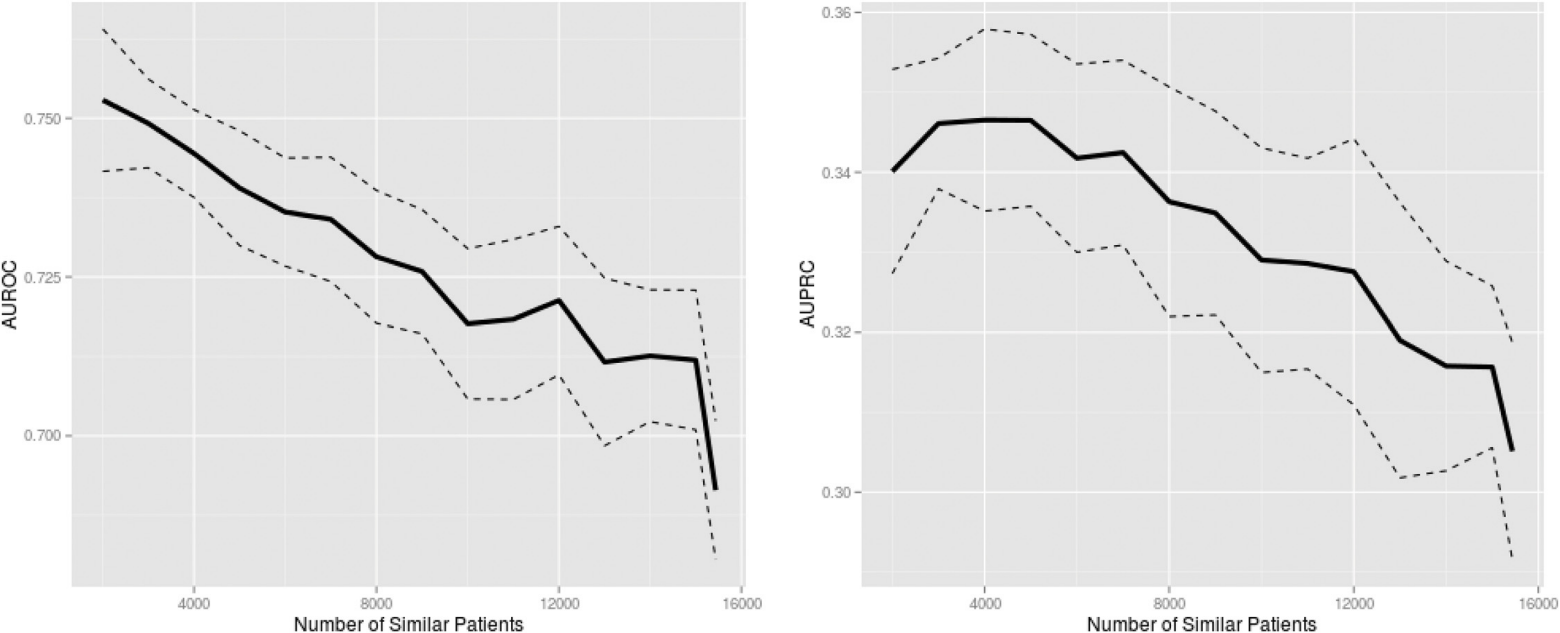
Mortality prediction performance of personalized decision trees trained on similar patient data. The solid and dashed lines are the mean and 95% confidence intervals, respectively, from 10-fold cross-validation. The maximum number of similar patients corresponds to all available training data. Predictive performance clearly improves as data from fewer but more similar patients are used for training. Identical outcome values in training data prohibited decreasing the number of similar patients further. AUROC: area under the receiver operating characteristic curve; AUPRC: area under the precision-recall curve.

Fig 3 depicts the relationship between the predictive performance of personalized DT and the number of similar patients used for training, while Table 4 tabulates the results shown in Fig 3. Fig 3 again supports our hypothesis that a smaller subset of similar patients comprises a better training dataset (i.e., both AUROC and AUPRC showed an increasing trend moving left on the X-axes).

**Table 4:** Mortality prediction performance of decision trees as a function of the number of similar patients used in training.

Number of Similar Patients	AUROC (Mean [95% CI])	AUPRC (Mean [95% CI])
2000	0.753 [0.742, 0.764]	0.340 [0.327, 0.353]
3000	0.749 [0.742, 0.756]	0.346 [0.338, 0.354]
4000	0.744 [0.738, 0.751]	0.347 [0.335, 0.358]
5000	0.739 [0.730, 0.748]	0.346 [0.336, 0.357]
6000	0.735 [0.727, 0.744]	0.342 [0.330, 0.354]
7000	0.734 [0.724, 0.744]	0.342 [0.331, 0.354]
8000	0.728 [0.718, 0.739]	0.336 [0.322, 0.351]
9000	0.726 [0.716, 0.736]	0.335 [0.322, 0.348]
10000	0.718 [0.706, 0.729]	0.329 [0.315, 0.343]
11000	0.718 [0.706, 0.731]	0.329 [0.315, 0.342]
12000	0.721 [0.710, 0.733]	0.328 [0.311, 0.344]
13000	0.712 [0.698, 0.725]	0.319 [0.302, 0.336]
14000	0.713 [0.702, 0.723]	0.316 [0.303, 0.329]
15000	0.712 [0.701, 0.723]	0.316 [0.306, 0.326]
15649	0.691 [0.680, 0.702]	0.305 [0.292, 0.319]

The results shown in Fig 3 are tabulated here. AUROC: area under the receiver operating characteristic curve; AUPRC: area under the precision-recall curve; CI: confidence interval.

The maximum AUROC of 0.753 and AUPRC of 0.347 were achieved with 2000 and 4000 most similar patients, respectively. These peak performances significantly outperformed the model that used all available training data (AUROC: p<10^–4^; AUPRC: p = 0.003). Also, the peak performances were significantly better than the SAPS (AUROC: p<10^–9^; AUPRC: p<10^–5^) and SOFA (AUROC: p<10^–11^; AUPRC: p<10^–4^) performances.

Although AUROC continued to improve as the number of similar patients approached the minimum of 2000, the peak AUPRC occurred at 4000 similar patients and small sample size effects were evident for AUPRC when fewer than 4000 similar patients were included in training. Furthermore, similarly to the LR results in Fig 2, there was a noticeable rapid decay in performance when the most dissimilar patients were added (the right ends of the plots in Fig 3, moving right from 15000 on the X-axes).

## Discussion

While personalized medicine is often discussed as an application of genome science, increasingly vast and accessible EMR repositories enable personalization based on other types of biomedical Big Data as well. In this study, we investigated the utility of archival clinical data in personalizing risk stratification in the ICU. The results confirmed our two major hypotheses, that 1) using a subset of similar patients rather than a larger, heterogeneous population as training data improves mortality prediction performance at the patient level, and 2) as fewer but more similar patients are used to train predictive models, performance improves initially due to increased homogeneity in training data but subsequently decreases due to small sample size effects. In our experiment, LR achieved the best performance while DT resulted in the worst. Of note, simple death counting among only 60–100 similar patients resulted in good predictive performance.

For individual clinical encounters, a prediction about the likelihood of a poor outcome is traditionally arrived at using two approaches in combination. First, the clinician may draw upon evidence from the medical literature, whether in the form of observational studies of patients with similar clinical features or SOI scores, to formulate an initial estimate of risk. Second, this estimate may then be adjusted upward or downward based on the judgment of the practitioner, taking into consideration prior cases seen in his or her practice that had similar features.

We propose a method to formalize the intuitive, common sense practice of basing decisions about an index case on past cases with similar features. This approach aims to capture some of the pattern recognition heuristics that inform diagnosis and prognostication in clinical medicine, while conferring a number of advantages over traditional evidence-based risk stratification. Most notably, PSM-based prediction outperformed not only widely used SOI scores but also customized models that used all available data for training. What’s more, PSM-based prediction is rooted in objective mathematical calculation drawing on reliable, immutable data. As such it can overcome biases that may encumber heuristic methods, such as recall bias, “last case” bias, and errors of fixation. Varying times spent in clinical practice as well as varying recall abilities also mean that the reliability of prior clinical exposure will vary substantially between practitioners. Unlike intuitive case-matching done on an individual basis, PSM-based mortality prediction is fully transparent, explicit, and objective, and can be used by practitioners in any career stream, and at any stage of practice.

Our results have implications beyond mortality prediction in intensive care. PSM-based methods could be easily adapted to predict other outcomes (e.g., length of hospital stay, re-admission to hospital) in a variety of medical fields. Extensions of PSM-based predictions could also be used to estimate the likelihood that a patient will respond (favorably or unfavorably) to a given therapeutic intervention. The ability to predict such “intermediate outcomes”, which may occur infrequently, is another domain in which PSM-based methods may be of use.

Deploying personalized decision support of this kind at the point of care requires an electronic repository of past clinical cases, computing resources that can efficiently calculate PSMs and train personalized predictive models, and a graphical user interface that can facilitate user input and display of decision support information. While these represent considerable resource challenges in a number of practice settings, the rising prevalence of electronic medical record (EMR) systems and modern information technology (IT) are bringing real-time personalized decision support increasingly within reach. While large, heterogeneous, multi-center patient data could be used as an alternative for decision support and prognostication, data from a local EMR are likely of greater value since institution-based patient similarities—including demographics, epidemiology, and practice patterns—are more accurately portrayed in local data. This point adds value to costly EMR adoption and opens opportunities for meaningful secondary use of EMR data.

Our study has a few limitations that bear further discussion. First, patients with missing data were excluded, which may have introduced selection bias. However, whether the included patient cohort was a fair representation of the entire MIMIC-II population was largely irrelevant to testing our hypotheses. In the era of EMR systems that conveniently archive large-scale data, patient data are abundant and exclusion of cases with missing data is an appropriate and viable option. Second, the PSM used in this study was restricted by the availability of variables in the MIMIC-II database. Although there will always be unobserved clinical and demographic features, inclusion of additional, complementary variables may further improve the PSM. Third, the improved performance of personalized prediction comes at the cost of increased computational burden. Instead of building and deploying a static, one-size-fits-all model, the proposed personalized decision support framework must dynamically compute pairwise PSM values, identify the most similar patients, build a custom model, and apply the model to the index patient. The computational burden depends on the complexity of the PSM calculation, and the size of the EMR database. Although further research is required to realize a fully automated, personalized clinical decision support system, modern IT has proven in other fields (e.g., e-commerce, personalized online advertising) that similar systems are feasible. In particular, the de-facto Big Data analytic platform, Apache Hadoop, can readily be deployed since pairwise PSM computation is very much parallelizable.

The present study employed a relatively intuitive, computationally inexpensive PSM. Many other PSMs could be generated by changing either the variables that comprise the PSM or the overall structure of PSM calculation. It is also possible that different medical specialties may want to use different PSM types. Future research in this area will focus on developing more complex and expressive PSMs, as well as enhancing the computational efficiency of matching algorithms, validating PSM-based prediction in other datasets, and testing these predictions prospectively.
